# Attitudes about involvement in hypothetical clinical trial protocols in Mexican and Mexican-American at risk for autosomal dominant Alzheimer’s disease

**DOI:** 10.1186/s13195-022-01114-4

**Published:** 2022-11-15

**Authors:** Angélica Zuno-Reyes, Esmeralda Matute, Karin Ernstrom, Mellissa Withers, Yaneth Rodriguez-Agudelo, Rema Raman, John M. Ringman

**Affiliations:** 1 Instituto de Neurociencias, CUCBA, Universidad de Guadalajara, Francisco de Quevedo 180, 44130 Guadalajara, Jalisco Mexico; 2 Departamento de Estudios en Educación, CUCSH, Universidad de Guadalajara, Guadalajara, Jalisco Mexico; 3 Department of Population and Public Health Sciences, Keck School of Medicine, University of Southern California, Los Angeles, CA USA; 4 Department of Neuropsychology, National Institute of Neurology and Neurosurgery, Mexico City, Mexico; 5 Alzheimer’s Disease Research Center, Department of Neurology, Keck School of Medicine at USC, Los Angeles, CA USA

**Keywords:** Autosomal dominant Alzheimer’s disease, Clinical trials, Genetic testing, Sociodemographic factors, Research recruitment, Mexicans, Mexican-Americans

## Abstract

**Background:**

The enrollment into clinical trials of persons at risk for autosomal dominant Alzheimer’s disease (ADAD) in whom the onset of disease can be accurately predicted facilitates the interpretation of outcomes (e.g., biomarkers, treatment efficacy). Attitudes toward involvement in such studies are biased by intrinsic cultural and social characteristics. Our objective was to study how demographic factors such as country of residence, age, sex, schooling, parenthood, and urbanization affect attitudes towards participation in hypothetical clinical trials in Mexican families at risk for ADAD living either in Mexico or in the United States.

**Methods:**

Participants were 74 members of different families known to harbor an ADAD mutation living in Mexico (*n* = 50) or in the United States (*n* = 24). Participants were asked, in a written questionnaire, their interest in participating in four hypothetical clinical trial scenarios of increasing perceived invasiveness. The questionnaire then asked about their willingness should there be a 50% chance of being assigned to a placebo group. The influences of demographic variables on decisions were performed using Wilcoxon rank-sum for continuous variables and Fisher’s exact test for categorical variables.

**Results:**

Participants who live in Mexico, who have or plan to have children, who do not attend or do not plan to attend school, and who live in rural areas gave more positive responses regarding their willingness to participate compared to those living in the U.S. The 50% chance of being in a placebo group increased the willingness to participate for family members living in Mexico. The main reason for participation was to help future generations, while the main reasons for refusal were not wanting to undergo genetic testing and consideration of adverse effects.

**Conclusions:**

We found a higher level of willingness to participate in clinical trials among persons living in rural Mexico and our data suggest that altruism towards future generations is a major motivation, though this was balanced against concerns regarding side effects. Our results emphasize the importance of sharing information and assessing its understanding in potential participants with diverse backgrounds in the nature of ADAD and regarding the design of clinical trials prior to their enrollment in such studies.

**Supplementary Information:**

The online version contains supplementary material available at 10.1186/s13195-022-01114-4.

## Background

Though some genetic variants contribute to disease risk in a probabilistic manner, others can be highly predictive of the development of disease. Information regarding the risks of inheriting a pathological condition has implications for both individuals with the disease and other members of his or her family network [1].

Autosomal dominant Alzheimer’s disease (ADAD) accounts for approximately 1% of all Alzheimer’s disease (AD) cases [2] and is usually caused by mutations in one of three known genes, namely *PSEN1* (OMIM 104311), *APP* (OMIM 104760), and *PSEN2* (OMIM 1600759). These mutations are essentially fully penetrant with a relatively consistent early age of onset within families and within mutations [3, 4]. The term early-onset refers to the age at which symptoms arise, which varies among mutation types and families [2], but typically occurs with the onset of symptoms before the age of 65 years. The age at symptom onset therefore has a direct impact on the patient’s productive years so that many of those affected and their potential caregivers (usually the partner of the affected person) tend to be employed, have active social lives, and have children living at home. Therefore, the disease can impact all aspects of the lives of affected persons and their family members [5].

There are currently no definitively effective disease-modifying treatments for either familial or sporadic Alzheimer’s disease [2]. Nonetheless, the enrollment of persons at risk of ADAD in clinical trials is a reliable method to assess the efficacy of interventions, be they for the prevention of symptom onset, or disease-modifying once symptoms have begun. While this is a strength in that it enables patients to be followed throughout the disease trajectory, few clinical trials are conducted in ADAD populations due to methodological (e.g., how to measure efficacy in presymptomatic persons) and ethical challenges (e.g., assignment of patients to the placebo arms, risk of inadvertent undesired disclosure of a genetic status that was not intended to be known) [6, 7].

In the United States (U.S.), ethnic minorities (e.g., African-American, Hispanic, and non-Hispanic) are less willing to participate in biomedical studies due to obstacles such as lack of trust, stigmas, and competing demands on their time [8]. However, this apparent underrepresentation may also be due to other social factors, including limited access to research opportunities, reduced invitations to participate, and specific attitudes towards genetic research, among others. This unbalanced participation biases research findings, limiting their generalizability to disenfranchised populations [8, 9]. These challenges to equitable and informed enrollment are further complicated among persons at risk for being ADAD mutation carriers due to a lack of understanding about the causes of AD, understanding of the rationale behind the methodological designs in studies, and the understanding of the nature of genetic risk [10, 11].

Previous studies assessing the desire to participate in genomic research on complex diseases recruiting African-American, Hispanic, and non-Hispanic white individuals show that the main justification for participation is altruistic reasons, to benefit family members, personal health benefits, personal curiosity, and to improve understanding of genetic mechanisms. Reasons for non-participation were a negative perception about the research, subjectively perceived lack of relevance, negative feelings about the medical procedures involved, and fear of the results [12].

Numerous families whose origins can be traced to Jalisco state, Mexico, have been identified as harboring the Ala431Glu mutation (A431E) in exon 12 of the *PSEN1* gene. Age of onset ranges from 34 to 53 years [4, 13, 14], making them appropriate for clinical trials. Yescas et al. [14] identified nine Mexican families with this mutation in their original report, while Murrell et al. [13] found another 15 independent families, all with ancestry coming from the state of Jalisco. Following these reports, Dumois-Petersen et al. [4] identified additional A431E cases including a genealogical analysis with 301 affected relatives of the mutation carriers (195 already deceased) and 560 descendants at 50% risk of carrying the mutation. Such a relatively large population provides the opportunity for performing clinical trials, albeit in a region in which presymptomatic genetic counseling and testing are not widely available. Also, no reliable estimates of the prevalence of ADAD in other regions of Mexico are available.

Our aim in this study was to determine how demographic factors including country of residence, age, sex, schooling, parenthood, and rural or urban residence affect attitudes towards genetic testing and clinical trials in Mexican families at risk of ADAD living either in Mexico or in the U.S.

## Methods

### Participants

Seventy-four participants who were members of families known to harbor an ADAD mutation, whose ancestry was traced to Jalisco, and whose residence is in California (*n* = 24), Jalisco (*n* = 43), or Mexico City (*n* = 7) were included. All the respondents gave written or verbal informed consent, the latter in a few of illiterate participants. A population density of less than 2500 inhabitants was considered to determine that a segment of the sample came from a rural locality [15].

Subsequently, they all attended a 90-min in-person presentation in which information about Alzheimer’s disease and the genetics underlying ADAD, and participation in ADAD-oriented research, including clinical trials were presented. The information was given by a neurologist with a clinical and research focus on ADAD (author J.R.).

This study was approved by the Institutional Review Board at the University of California, Los Angeles, The University of Southern California, the National Institute of Neurology and Neurosurgery in Mexico City, and the University of Guadalajara.

### Materials

The written questionnaire was administered in English or Spanish to all participants depending on their preferred language. The questionnaire consists of four hypothetical scenarios of preventive interventions with increasing levels of invasiveness and health risks, where the participant is required to undergo a genetic test as an inclusion criterion. In order to address the questions of invasiveness and risk, the hypothetical studies were short simplifications modeled after currently ongoing trials in AD (e.g., oral medication, vaccination trials). We sought to query a range of study designs without overwhelming participants with details. Participants indicated their willingness or their denial to undergo genetic testing and participate in hypothetical trials based on preset answers (e.g., yes — to help future generations, no — the risks and side effects are too high to justify possible benefits), or with the alternative of choosing “other” and elaborate a statement of their reasons. Multiple responses could justify the inclination to participate or not to participate in the hypothetical clinical trials. Subsequently, for each trial design, their willingness to participate if there was a 50% chance of receiving a placebo was explored. In addition to the questionnaire on hypothetical clinical trial scenarios, participants completed two questionnaires, the Research Attitudes Questionnaire (RAQ-7), which asks about attitudes towards medical research, and the Genetic Knowledge Questionnaire (GenQuest), which asks about knowledge of genetics in AD. These questionnaires were not analyzed for this study, but we include the results as supplementary information. Participants were given ample opportunity to ask questions during the informational sessions and when completing the questionnaires, with answers provided by members of the research team.

#### *Hypothetical study 1 (H1)*

Study 1 read as follows: “An institution is looking for participants for a research study for a medication with substantial promise in preventing AD. The medication has been studied extensively in animals and humans and is felt to be very safe. The treatment is a pill, taken twice a day that would most likely be required for the rest of your life.”

#### *Hypothetical study 2 (H2)*

Study 2 read as follows: “A research study is looking at the effects of a vaccination that is given once per year for the rest of your life and hopefully will provide protection from the development of AD. Earlier studies of this vaccination in people have shown a 5% risk of brain inflammation that leads to permanent neurological disability (like a stroke) in 1% of subjects.”

#### *Hypothetical study 3 (H3)*

Study 3 read as follows: “A drug company wants to test a medication that would be administered intravenously every three months for the rest of persons’ lives. Similar to the vaccination study, prior research in people has shown a 5% risk of brain inflammation that leads to permanent neurological disability (like a stroke) in 1% of subjects.”

#### *Hypothetical study 4 (H4)*

Study 4 read as follows: “A research study is looking for participants for a high-risk clinical trial involving brain surgery. In this study, a neurosurgeon would drill small holes, one on each side of your skull while you are asleep under anesthesia. They would then implant a substance directly into your brain. You would only have to undergo this procedure once in your lifetime. The risks of the surgery and anesthesia can be high, and may include death. Results cannot be guaranteed. However, if the treatment worked, you would not develop AD or it would develop later in life than you would have otherwise.”

### Data analysis

Participant’s demographic characteristics, questionnaires, and willingness to participate in clinical trials including their reasons are summarized by country of residency; frequencies and percentages were included for categorical variables; continuous variables are summarized with mean and standard deviation (SD). Participant’s demographic characteristics were compared using Wilcoxon rank-sum for continuous variables and Fisher’s exact test for categorical variables. To analyze the influence of age, we defined two groups according to the median age of the overall sample given the small sample size, the age distribution of the participants, and to better reflect their sociodemographic characteristics. The median age of the overall sample is 37 (27–44) years, while the median age of participants living in Mexico is 38.5 (31.2–46.5) years and 35 (22.8–38.8) years for participants living in the U.S.; thus, we formed two age subgroups; one was composed of participants aged 37 years or older and the other with less than 37 years. We replicated the procedure for the analysis of the influence of years of schooling using the same rationale as the subgrouping for age based on the median, where the overall median was 12 (9–15) years of schooling, while the median schooling for participants living in Mexico was 11 (9–13) years and 13 (12–15) years for participants living in the U.S.; thereafter, we formed two subgroups, one with 12 or more years of schooling and the other with less than 12 years of schooling. Not all participants responded to all hypothetical scenarios about their willingness to participate nor with regard to their reasons for participating or not participating. All responses that were provided were included in the analysis. Willingness to participate in clinical trials was summarized by country of residency, sex, school attendance, parenthood, living setting according to degree of urbanization, age (median split), and years of schooling (median split). The influence of demographic variables on the willingness to participate in clinical trials was performed using Wilcoxon rank-sum for continuous variables and Fisher’s exact test for categorical variables. For the participant’s data with respect to the influence of school attendance, both currently attending and planning on attending further schooling were considered together for the analysis in light of statistical power considerations. Participants comprising the category “planning on attending further schooling” (*n* = 20) include both participants who are currently attending school (*n* = 8, 40%) and participants who do not attend but plan to do so in the future (*n* = 12, 60%).

For the parenthood analysis, those participants who have and those who plan to have children were considered together as well, again due to statistical power considerations. Participants who “plan to have children” (*n*=16) include both those who already have descendants (*n*=7, 44%) and those who do not currently have children but plan to have in the future (*n*=9, 56%). Statistical analysis was done using the statistical software R (www.r-project.org). A *p* value <0.05 was considered statistically significant.

## Results

### Demographic

A total of 74 participants aged 18–71 years old (mean 36.5, SD = 11.8), of which 50 live in Mexico and 24 in the United States (U.S.) were part of this study (Table 1). According to their self-report, all participants living in Mexico, but only 2 participants living in the U.S. speak Spanish as a preferred language; in both countries, most participants were women (68.9%). We found that among the participants living in Mexico, 52% were living in rural areas (52%), while only one of those living in the U.S. was living in a rural area. Not only the average number of years of schooling was lower in the group of participants living in Mexico (2.5-year difference), but also this group show a greater variability (SD 4.2 vs. 2.1) in the number of years of education completed.

**Table 1:** Demographic information of the participants at risk of ADAD living in Mexico or in the U.S.

	Mexico (*n*=50)	U.S. (*n*=24)	Overall (*n*=74)	*p value*
No. of females (%)	33 (66)	18 (75)	51 (68.90)	0.593
Age (SD)	38.20 (12.38)	32.83 (9.63)	36.5 (11.80)	0.068
Years of schooling (SD)	11.10 (4.18)	13.61 (2.06)	11.9 (3.80)	0.003
Speak Spanish as the preferred language (%)	50 (100)	2 (8.33)	52 (70.30)	<0.001
Living in urban/suburban (%)	24 (48)	23 (95.83)	47 (63.50)	<0.001
Currently attending school (%)	8 (16)	8 (33.33)	16 (21.60)	0.131
Plan on attending further school (%)	13 (28.3)	7 (35)	20 (30.3)	0.576
Have children (%)	34 (70.83)	14 (58.33)	48 (66.7)	0.303
Plan to have children (%)	7 (15.55)	9 (39.13)	16 (23.50)	0.039

The variables age and years of schooling are shown as mean and in parentheses, standard deviation

*ADAD* autosomal dominant Alzheimer’s disease, *U.S.* United States, *SD* standard deviation

### Willingness to participate in ADAD hypothetical clinical trial protocols

Of the 74 participants, 72 (97%) answered H1, 70 (95%) answered H2 and H3, and 67 (90%) answered H4. Willingness to participate (coded as a “yes” answer) in hypothetical research protocols and clinical trials of treatments for ADAD was analyzed by country of residence, sex, current and the planned attendance of school, actual parenthood and the plan to have more children, rural or urban living situation, age, and years of schooling achieved (Table 2). Regarding the country of residence, we found differences only in the willingness to get involved in *Hypothetical study 1* (oral medication protocol), with greater acceptance by participants living in Mexico compared to those living in the U.S. (92% vs. 61%, *p* = 0.003). When considering the influence of school attendance on the willingness to get involved in the hypothetical studies, participants who currently attend school showed lower acceptance to *Hypothetical study 4* (brain surgery protocol), than those who do not attend school (41% vs. 11%, *p* = 0.012). Among the participants who have/plan or do not have/plan to have children, we found significant differences only in the clinical trials with vaccines (H2) and in the administration of intravenous medication (H3). In both cases, participants with children or planning to have children were more likely to be willing to participate than those without children (74% vs. 33%, *p* = 0.006). In the scenario of the intravenous drug trial, the result was consistent with the previous trend, with greater acceptance by the population with children than in the population without progeny (70% vs. 25%, *p* = 0.003).

**Table 2:** Willingness to participate in the four hypothetical protocols according to our target variables

**I. Country of residency**				
	Mexico (*n* = 50)	U.S. (*n* = 24)	Overall (*n* = 74)	*p value*
H1: Oral medication trial	45 (92%)	14 (61%)	59 (82%)	0.003
H2: Vaccine trial	34 (71%)	12 (55%)	46 (66%)	0.278
H3: Intravenous drug trial	33 (67%)	9 (43%)	42 (60%)	0.067
H4: Neurosurgery trial	16 (35%)	3 (14%)	19 (28%)	0.143
**II. Gender**				
	Female (*n* = 51)	Male (*n* = 23)	Overall (*n* = 74)	*p value*
H1: Oral medication trial	40 (82%)	19 (83%)	59 (82%)	1
H2: Vaccine trial	30 (62%)	16 (73%)	46 (66%)	0.588
H3: Intravenous drug trial	27 (56%)	15 (68%)	42 (60%)	0.434
H4: Neurosurgery trial	14 (30%)	5 (24%)	19 (28%)	0.771
**III. Schooling**				
	Currently not attending/planning more school (*n* = 46)	Currently attending/planning more school (*n* = 28)	Overall (*n* = 74)	*p value*
H1: Oral medication trial	37 (84%)	22 (79%)	59 (82%)	0.55
H2: Vaccine trial	31 (74%)	15 (54%)	46 (66%)	0.123
H3: Intravenous drug trial	29 (69%)	13 (46%)	42 (60%)	0.082
H4: Neurosurgery trial	16 (41%)	3 (11%)	19 (28%)	0.012
**IV. Parenthood**				
	Do not have or plan to have children (*n* = 16)	Do have or plan to have children (*n* = 57)	Overall (*n* = 73)	*p value*
H1: Oral medication trial	11 (73%)	47 (84%)	58 (82%)	0.452
H2: Vaccine trial	5 (33%)	40 (74%)	45 (65%)	0.006
H3: Intravenous drug trial	4 (25%)	37 (70%)	41 (59%)	0.003
H4: Neurosurgery trial	4 (27%)	15 (29%)	19 (29%)	1
**V. Living setting according to the degree of urbanization**				
	Rural (*n* = 27)	Urban/suburban (*n* = 47)	Overall (*n* = 74)	*p value*
H1: Oral medication trial	27 (100%)	32 (71%)	59 (82%)	0.001
H2: Vaccine trial	25 (93%)	21 (49%)	46 (66%)	< 0.001
H3: Intravenous drug trial	25 (93%)	17 (40%)	42 (60%)	< 0.001
H4: Neurosurgery trial	13 (50%)	6 (15%)	19 (28%)	0.002
**VI. Age (median split)**				
	< 37 years (*n* = 34)	> 37 years (*n* = 40)	Overall (*n* = 74)	*p value*
H1: Oral medication trial	26 (79%)	33 (85%)	59 (82%)	0.554
H2: Vaccine trial	24 (73%)	22 (59%)	46 (66%)	0.315
H3: Intravenous drug trial	20 (61%)	22 (59%)	42 (60%)	1
H4: Neurosurgery trial	8 (24%)	11 (32%)	19 (28%)	0.590
**VII. Years of schooling (median split)**				
	< 12 years (*n* = 29)	> 12 years (*n* = 44)	Overall (*n* = 73)	*p value*
H1: Oral medication trial	28 (97%)	30 (71%)	58 (82%)	0.010
H2: Vaccine trial	25 (86%)	20 (50%)	45 (65%)	0.002
H3: Intravenous drug trial	24 (83%)	17 (42%)	41 (59%)	0.001
H4: Neurosurgery trial	12 (44%)	7 (18%)	19 (29%)	0.028

H1, H2, H3, and H4, each refers to the four different hypothetical protocols. *U.S.* United States. Age and years of schooling variables were categorized by separating those below and above or equal to the median

In the comparison by living setting (rural or urban), we found that participants living in rural areas have a greater willingness to participate in all protocols compared to participants living in urban areas. This result is replicated in those participants whose years of schooling corresponding to our sample were below the median (11 years of schooling) compared to participants above the median. We found no differences according to sex or age.

### Reasons for participation and non-participation in the ADAD hypothetical clinical trial protocol

In all hypothetical research protocols proposed, we found that the reason to “help future generations” was the main reason chosen for justifying participation (Table 3), both for participants living in Mexico (87%) and those living in the U.S. (93%).

**Table 3:** Reasons to participate and not to participate in the four hypothetical protocols

**H1: Oral medication**		
**Reasons to participate**	**Mexico (*n*=45)**	**U.S. (*n*=14)**
Benefits outweigh the risk	30 (67%)	10 (71%)
Help future generations	39 (87%)	13 (93%)
Other	0 (0%)	1 (7%)
**Reasons to decline participation**	**(*n*= 4)**	**(*n*=9)**
Do not want to know my genetic status	1 (25%)	5 (56%)
Risks and side effects are too high to justify possible benefits	1 (25%)	2 (22%)
Other	2 (50%)	3 (33%)
**H2: Vaccine trial**		
**Reasons to participate**	**(*n*=34)**	**(*n*=12)**
Benefits outweight the risk	21 (62%)	8 (67%)
Help future generations	25 (74%)	8 (67%)
Other	1 (3%)	1 (8%)
**Reasons to decline participation**	**(*n*=14)**	**(*n*=10)**
Do not want to know my genetic status	0 (0%)	5 (50%)
Risks and side effects are too high to justify possible benefits	12 (86%)	4 (40%)
Other	1 (7%)	0 (0%)
**H3: Intravenous drug trial**		
**Reasons to participate**	**(*n*=33)**	**(*n*=9)**
Benefits outweight the risk	22 (67%)	7 (78%)
Help future generations	28 (85%)	7 (78%)
Other	1 (3%)	0 (0%)
**Reasons to decline participation**	**(*n*=16)**	**(*n*=12)**
Do not want to know my genetic status	4 (25%)	7 (58%)
Risks and side effects are too high to justify possible benefits	9 (56%)	4 (33%)
Other	3 (19%)	1 (8%)
**H4: Neurosurgery**		
**Reasons to participate**	**(*n*=16)**	**(*n*=3)**
Benefits outweight the risk	10 (62%)	2 (67%)
Help future generations	15 (94%)	2 (67%)
Other	0 (0%)	0 (0%)
**Reasons to decline participation**	**(*n*=30)**	**(*n*=18)**
Do not want to know my genetic status	3 (10%)	7 (39%)
Risks and side effects are too high to justify possible benefits	23 (77%)	11 (61%)
Other	5 (17%)	3 (17%)

H1, H2, H3, and H4, each refers to the four different hypothetical protocols respectively. *U.S.* United States

Overall, 22% of the 59 participants who gave reasons to participate or not in H1 indicated they did not wish to be tested to participate in a study of oral medication. Among the reasons for not participating in H1, we found that the option “I don’t want to know my genetic status” was the most selected response (46%), with specifications such as “I prefer not to know my ADAD status,” “At this point, I would not be able to handle the stress of knowing if I undoubtedly carry the ADAD gene,” “I don’t want to stress myself,” and “I would not like to think about it and live what I have left, I would just like to control it (the disease) without knowing I have the gene” given by the participants. In the case of protocols involving the application of a vaccine, intravenous medication, or brain surgery, 34%, 40%, and 72% of persons respectively indicated they did not want to participate.

Among the reasons, the “Risks and side effects are too high to justify possible benefits” answer was the most frequently selected. For the neurosurgery trial, participants provided answers such as “It is too dangerous for me and sounds painful,” “extremely dangerous,” “I do not want to undergo brain surgery,” “not 100% safe,” and “because I would not like to die.”

### Willingness to participate if there was a 50% chance of receiving placebo in each hypothetical protocol

We found that in participants living in Mexico, including a 50% chance of being assigned to the placebo group in the research protocol increases the desire to participate in clinical trials involving vaccine (90% with placebo vs. 71% with no placebo), intravenous drug (83% with placebo vs. 67% with no placebo), and neurosurgery (75% with placebo vs. 35% with no placebo), but not for the oral medication protocol (88% with placebo vs. 92% with no placebo). The opposite happens in participants living in the U.S., for whom the probability of receiving placebo decreases their desire to be part of the oral medication (45% with placebo vs. 61% with no placebo), vaccine (48% with placebo vs. 55% with no placebo), and intravenous medication (30% with placebo vs. 43% with no placebo) protocols, except for the neurosurgery trial (19% with placebo vs. 14% with no placebo). The overall percentage that includes participants living in Mexico and in the U.S. (Table 4) indicates that the vaccine trial is the one with the highest acceptance, while the protocol involving neurosurgery is the one with the lowest acceptance.

**Table 4:** Willingness to participate in the four hypothetical protocols if there was a 50% chance of receiving a placebo

	Combined Mexico and U.S. samples (*n* = 74)
H1: Oral medication trial, 50% placebo	51 (75%)
H2: Vaccine trial, 50% placebo	54 (77%)
H3: Intravenous drug trial, 50% placebo	46 (68%)
H4: Neurosurgery trial, 50% placebo	37 (57%)

H1, H2, H3, and H4, each refers to the four different hypothetical protocols respectively. *U.S.* United States

### Reasons for participation and non-participation in the ADAD hypothetical clinical trial protocols when a 50% chance of receiving placebo is included

For the first protocol with oral medication, the prevailing reason to justify participation was because the “benefits outweigh the risks” with 80% of all participants choosing this response. The most common reason for participation in the other hypothetical studies both for participants living in Mexico and in the U.S., even when there was a 50% chance of receiving placebo was to “help future generations.”

As for the reasons for not participating, in the oral medication protocol, the most frequently selected reason was the risk of knowing their genetic status and with other explanations such as “because I want the medication” when there was a placebo involved. In the protocol involving the administration of a vaccine, not wanting to know the genetic status and thinking that the risks and side effects were too high were the most selected responses (44% each). For intravenous drug administration and in the brain surgery study, the most frequently selected option (55% and 61%, respectively) was that the risks and side effects are too high. Specific reasons such as “being vaccinated every 3 months for the rest of my life would be hard for me and I would be scared of side effects” and “This whole procedure seems dangerous and painful and I’d rather not” were enunciated.

## Discussion

The main purpose of this study was to explore the attitudes about genetic testing and clinical trials in persons of Mexican ancestry at risk for ADAD mutations in relation to country of residence, age, sex, years of schooling, current school attendance, parenting plans, and living situation (rural or urban). Having a majority of participants living in Mexico and speaking Spanish was as expected, given that family groups at risk of carrying a determinant ADAD mutation whose origins are traced to Jalisco have been identified [4, 13, 14]. Specifically, these Mexican families are concentrated in both rural and some urban areas, while a subset has migrated to the U.S. as part of a common practice of Mexican labor migration.

Understanding the nature of ADAD and its inheritance is fundamental to the ethical conduct of clinical trials and recruitment of persons at risk for ADAD. Specifically, an understanding of the methodological aspects of studies and their duration, the rights of research participants, available treatments, procedures, and risks are critical to informed consent [15]. However, this understanding can be compromised by demographic factors such as literacy level, years, and quality of education [16]. We found that the group who does not attend school currently or whose educational level is below the sample’s median had greater acceptance of participation in all the protocols. This may reflect a different understanding of the proposed procedures, risks, and potential benefits or an increased degree of trust towards biomedical research. It will be crucial to inquire further about the conditions behind the motivation to participate in people with fewer years of schooling before assuming that this is due to a lower or higher understanding of the information presented.

Consistent with previous results on attitudes towards clinical trials and research protocols on Latino families at risk of ADAD mutations [10, 12, 16], we found that the most frequent reason to justify participation was altruism, “to help future generations.” This was supported by analyzing separately the frequency of acceptance of those with/planning to have children, where it was more frequent to accept participation in all protocols compared to the group without children, especially, when the hypothetical trials included vaccines and intravenous drugs.

As for the main reason for not participating in the hypothetical protocols, the risk of knowing one’s genetic status was the most frequently selected. Previous studies on the attitudes toward learning individual genetic status reveal that Hispanic and African-American participants expressed a preference to undergo genetic testing, but in turn, know less about these medical procedures than non-Hispanic white groups tested [17]. Specifically, groups of Mexican families where one of their members developed ADAD expressed interest in knowing their status [10, 16]. This poses a challenge considering that genetic counseling services are not always readily available, particularly in Mexico, where the responsibility falls on medical geneticists [16]. As a result, presymptomatic testing is rarely performed.

As the degree of invasiveness of the hypothetical interventions progressed, the number of participants who responded decreased from 72 (97%) willing to participate in the first study to 67 (90%) participants willing to participate in the fourth study. When there was a 50% possibility of receiving placebo, we found that participants living in Mexico increased their intention to enroll in the hypothetical protocols. A prior study carried out by our group of persons at risk for ADAD living either in the U.S. or in Mexico and non-Latino Caucasians showed that the probability of enrolling in a clinical trial and knowing one’s genetic status in a trial of an oral medication described as “safe” decreases when there was a possibility of receiving placebo [10], but does not in studies perceived to be of higher risk. Though our current results could be due to an incomplete understanding of placebo, the fact that more persons in Mexico were interested in participating in studies of higher risk interventions that featured a placebo arm suggests they understand the nature of placebo and that altruism may play an important role.

Withers et al. [16] surveyed and interviewed 123 family members of Mexican ADAD patients living either in Mexico or the U.S. about cultural beliefs surrounding dementia and participation in clinical trials. The participants reported receiving little or no information from healthcare providers about Alzheimer’s disease or its implications. They confirmed that family members of Mexican patients with ADAD have a lack of medical and scientific information about both AD in general and ADAD specifically. The authors suggest that members of these families may not receive information about the disease from healthcare providers or that there are educational, linguistic, and cultural barriers that make it difficult for them to understand the information presented. This lack of a comprehensive understanding of the disease and its inheritance indicates there are still challenges to overcome before performing ethical clinical trials to prevent ADAD.

### Limitations

Given the relatively small sample size and that the group of participants living in Mexico is twice as large as those living in the U.S., as well as the intrinsic cultural differences of each group, our data should be interpreted more as a descriptive report regarding the experience of these families rather than as a comprehensive analysis in which the independent effect of dependent variables can be disentangled.

The responses obtained by the surveyed participants reflect a potential interest in participating in hypothetical studies, rather than a measure of actual participation in research. More data regarding real-life scenarios where families can participate will allow us to delve more deeply into motives around enrollment in such studies. In this sense, the hypothetical scenarios we describe are based on real medical interventions, but do not include specific details regarding study protocols (e.g., study duration, time between assessments, inclusion criteria, techniques used such as neuropsychological testing, imaging studies, cerebrospinal fluid sampling) of active clinical trials. Though they therefore represent oversimplifications, provision of further details would likely have overwhelmed study participants. Future studies might explore attitudes regarding specific study procedures beyond the necessity of undergoing genetic testing (e.g., lumbar punctures, nuclear imaging, duration of study), perceived invasiveness, and the effects of placebo, rather than about study protocols as a whole.

Nonetheless, the inquiry of attitudes is of interest for the future implementation of actual protocols, as we have learned it will be important to generate strategies to improve communication regarding genetic information to ensure participants’ understanding of the implications of inheriting such mutations, as well as aspects of the onset and trajectory of ADAD and the protocol procedures. Likewise, upon finding differences in the intention to enroll in the hypothetical protocols according to years of schooling, it will be necessary to develop appropriate educational approaches and materials, particularly in rural areas with limited access to such information.

As our sample consists of a specific population at risk for a rare familial condition, we recommend discretion when interpreting our results, as they may not represent the attitude of other people in other geographic and cultural situations about participating in other biomedical protocols.

Finally, the survey consisted of multiple-choice answers, with the opportunity to delve more deeply into specific attitudes in each of the hypothetical protocols. For future studies with these families, we recommend the use of an instrument that more carefully measures the understanding of the placebo concept and additional methodological aspects of clinical trials, such as genetic testing and disclosure of genetic results; procedures for obtaining biomarkers, such as lumbar puncture, nuclear imaging, and other medical aspects; the length and timing of evaluation appointments; and even the understanding of the genetic causes and natural history of ADAD.

## Conclusions

Our results show a distinctive pattern of responses about participating in hypothetical ADAD clinical trials and research protocols depending on specific sociodemographic factors, between families with similar cultural features, but living in two different countries. We found a greater tendency to endorse participation by participants that live in Mexico, even among trials with an increased level of risk. This was particularly evident among those living in rural areas, with less or equal to 12 years or fewer of schooling, and those who have or plan on having children. No difference was observed in the acceptance to participate based on age or sex. The main reason given for enrollment was for altruistic reasons and to help future generations, while the main reason for refusing to participate was because they did not want to find out about their genetic status or because the risks of the procedures were too high. Interestingly, the sample living in Mexico was more interested in participating when a 50% chance of being assigned to a placebo group was involved. Further probing of these findings will be necessary to clarify whether people understand the concept and implementation of the placebo group and, second, to better understand the motivations behind the decisions expressed by the participants and its relation to intrinsic sociodemographic intrinsic factors within these families.

## Supplementary Information

**Additional file 1: Table S1.** Scores obtained on the GenQuest and RAQ questionnaires for both ADAD family members samples.

## Data Availability

The datasets used and/or analyzed during the current study are available from the corresponding author on reasonable request.

## Abbreviations

AD: Alzheimer’s disease
ADAD: Autosomal dominant Alzheimer’s disease
PSEN1: Presenilin 1
PSEN2: Presenilin 2
APP: Amyloid precursor protein
OMIM: Online Mendelian Inheritance in Man
U.S.: United States
RAQ-7: Research Attitudes Questionnaire
GenQuest: Genetic Knowledge Questionnaire
H1: Hypothetical protocol 1
H2: Hypothetical protocol 2
H3: Hypothetical protocol 3
H4: Hypothetical protocol 4
SD: Standard deviation
INEGI: Instituto Nacional de Estadística y Geografía

## References

[1] Andrews LB, Knoppers BM, Laberge C (1997). The genetic information superhighway: rules of the road for contacting relatives and recontacting former patients. Human DNA: law and policy: international and comparative perspectives.

[2] Pavisic IM, Nicholas JM, O'Connor A, Rice H, Lu K, Fox NC, Ryan NS (2020). Disease duration in autosomal dominant familial Alzheimer disease: a survival analysis. Neurol Genet.

[3] Ryman DC, Acosta-Baena N, Aisen PS, Bird T, Danek A, Fox NC, Goate A, Frommelt P, Ghetti B, Langbaum JB, Lopera F, Martins R, Masters CL, Mayeux RP, McDade E, Moreno S, Reiman EM, Ringman JM, Salloway S, Schofield PR, Sperling R, Tariot PN, Xiong C, Morris JC, Bateman RJ, Dominantly Inherited Alzheimer Network (2014). Symptom onset in autosomal dominant Alzheimer disease: a systematic review and meta-analysis. Neurology..

[4] Dumois-Petersen S, Gallegos-Arreola MP, Magaña-Torres MT, Perea-Díaz FJ, Ringman JM, Figuera LE (2020). Autosomal dominant early onset Alzheimer’s disease in the Mexican state of Jalisco: high frequency of the mutation PSEN1 c.1292C>A and phenotypic profile of patients. Am J Med Genet C: Semin Med Genet.

[5] Wattmo C, Wallin ÅK (2017). Early-versus late-onset Alzheimer disease: long-term functional outcomes, nursing home placement, and risk factors for rate of progression. Dement Geriatr Cogn Dis Extra.

[6] Ringman JM, Grill J, Rodriguez-Agudelo Y, Chavez M, Xiong C (2009). Commentary on “a roadmap for the prevention of dementia II: Leon Thal Symposium 2008.” Prevention trials in persons at risk for dominantly inherited Alzheimer’s disease: opportunities and challenges. Alzheimers Dement.

[7] Grill JD, Bateman RJ, Buckles V, Oliver A, Morris JC, Masters CL, Klunk WE, Ringman JM (2015). A survey of attitudes toward clinical trials and genetic disclosure in autosomal dominant Alzheimer’s disease. Alz Res Therapy.

[8] Ringman JM, Flores DL (2005). Earlier Alzheimer onset in Latino persons: ethnic difference vs. selection bias. Arch Neurol.

[9] Cuccaro ML, Manrique CP, Quintero MA, Martinez R, McCauley JL (2020). Understanding participation in genetic research among patients with multiple sclerosis: the influences of ethnicity, gender, education, and age. Front Genet.

[10] Hooper M, Grill JD, Rodriguez-Agudelo Y, Medina LD, Fox M, Alvarez-Retuerto AI, Wharton D, Brook J, Ringman JM (2013). The impact of the availability of prevention studies on the desire to undergo predictive testing in persons at risk for autosomal dominant Alzheimer’s disease. Contemp Clin Trials.

[11] Korenman S, Finder SG, Ringman JM (2015). Conceptualization and assessment of vulnerability in a complex international Alzheimer’s research study. Am J Bioeth.

[12] Sanderson SC, Diefenbach MA, Zinberg R, Horowitz CR, Smirnoff M, Zweig M, Streicher S, Wang Jabs E, Richardson LD (2013). Willingness to participate in genomics research and desire for personal results among underrepresented minority patients: a structured interview study. J Community Genet.

[13] Murrell J, Ghetti B, Cochran E, Macias-Islas MA, Medina L, Varpetian A, Cummings JL, Mendez MF, Kawas C, Chui H, Ringman JM (2006). The A431E mutation in PSEN1 causing familial Alzheimer’s disease originating in Jalisco State, Mexico: an additional fifteen families. Neurogenetics..

[14] Yescas P, Huertas-Vázquez A, Villarreal-Molina MT, Rasmussen A, Tusié-Luna MT, López M, Canizales-Quinteros S, Alonso ME (2006). Founder effect for the Ala431Glu mutation of the presenilin 1 gene causing early-onset Alzheimer’s disease in Mexican families. Neurogenetics..

[15] INEGI. Población rural y urbana. Población total según tamaño de la localidad para cada entidad federativa, 1950 - 2010. Censo de Población y Vivienda 2020. México: INEGI; c2022 Apr. Available from: https://cuentame.inegi.org.mx/poblacion/rur_urb.aspx?tema=P

[16] Withers M, Sayegh P, Rodriguez-Agudelo Y, Ernstrom K, Raman R, Montoya L, Zuno-Reyes A, Mosieri C, Matute E, Ringman JM (2019). A mixed-methods study of cultural beliefs about dementia and genetic testing among Mexicans and Mexican-Americans at-risk for autosomal dominant Alzheimer’s disease. J Genet Couns.

[17] Singer E, Antonucci T, Van Hoewyk J (2004). Racial and ethnic variations in knowledge and attitudes about genetic testing. Genet Test.

