# Supplementary material for: Attitudes about involvement in hypothetical clinical trial protocols in Mexican and Mexican-American at risk for autosomal dominant Alzheimer’s disease
Source: Alzheimers Res Ther. 2022 Nov 15;14:173. doi: 10.1186/s13195-022-01114-4 (PMC9664662; doi:10.1186/s13195-022-01114-4)
Supplement: Supplementary file 1 — Additional file 1: Table S1. Scores obtained on the GenQuest and RAQ questionnaires for both ADAD family members samples. [file 13195_2022_1114_MOESM1_ESM.docx]

**Supplementary Information:**

**Attitudes about involvement in hypothetical clinical trial protocols in Mexican and Mexican-American at-risk for autosomal dominant Alzheimer’s disease**

Angélica Zuno Reyes, M.S.^1^, Esmeralda Matute, Ph.D. ^1, 5^, Karin Enstrom, M.S.^3^, Mellissa Withers, Ph.D.^3^, Yaneth Rodriguez-Agudelo, Ph.D.^4^, Rema Raman, Ph.D.^3^, John M. Ringman, M.D. ^2^

1. Instituto de Neurociencias, CUCBA, Universidad de Guadalajara, Guadalajara, Jalisco, Mexico

2. Alzheimer's Disease Research Center, Department of Neurology, Keck School of Medicine at USC, Los Angeles, California, USA

3. Department of Population and Public Health Sciences, Keck School of Medicine, University of Southern California, Los Angeles, California, USA

4. Department of Neuropsychology, National Institute of Neurology and Neurosurgery, Mexico City, Mexico

5. Departamento de Estudios en Educación, CUCSH, Universidad de Guadalajara, Guadalajara, Jalisco, Mexico

Please send correspondance to: Angélica Zuno Reyes, M.S., Instituto de Neurociencias, Francisco de Quevedo 180, Guadalajara, JAL 44130, MEXICO. e-mail: angelicazunor@gmail.com

| **Table S1.** Scores obtained on the GenQuest and RAQ questionnaires for both ADAD family members samples. | | | | | |
| --- | --- | --- | --- | --- | --- |
|  |  | N | Mean | SD | *p value* |
| GenQuest (0 - 14) | Mexico | 36 | 11.5 | 2 |  |
|  | U.S. | 7 | 12.3 | 2.6 |  |
|  | Overall | 43 | 11.6 | 2.1 | 0.378 |
| RAQ (-17 to 17) | Mexico | 37 | 6 | 5 |  |
|  | U.S. | 5 | 6.4 | 2.3 |  |
|  | Overall | 42 | 6 | 4.7 | 0.984 |
| Note: ADAD: autosomal dominant Alzheimer's disease; U.S.: United States; GenQuest: Genetic Knowledge Questionnaire - higher scores indicating more knowledge about the genetics of AD; RAQ: Research Attitudes Questionnaire - more positive scores representing a more favorable attitude towards research; p-values are from Wilcoxon rand-sum test. | | | | | |
|  |  |  |  |  |  |
